# Stereoselective synthesis of (2*S*,3*S*,4*Z*)-4-fluoro-1,3-dihydroxy-2-(octadecanoylamino)octadec-4-ene, [(*Z*)-4-fluoroceramide], and its phase behavior at the air/water interface

**DOI:** 10.3762/bjoc.4.12

**Published:** 2008-04-25

**Authors:** Gergana S Nikolova, Günter Haufe

**Affiliations:** 1 Organisch-Chemisches Institut, Westfälische Wilhelms-Universität Münster, Corrensstr. 40, D-48149 Münster, Germany

## Abstract

**Background:**

Sphingolipids belong to the most important constituents of the membranes of eukaryotic cells. As intermediates in sphingolipid metabolism, sphingosine and its *N*-octadecanoyl-derivative, ceramide, exhibit a variety of biological functions. These compounds play a crucial role in many essential biological processes such as cell growth, cell differentiation, cell recognition and apoptosis. More specifically, sphingolipids are crucial e.g. for the function of the skin because they contribute to the formation of the water permeability barrier consisting of a highly organized multilaminar lipid matrix of free fatty acids, cholesterol and ceramides containing additional hydroxyl groups in the sphingosin part and longer fatty acid amide functions.

**Results:**

In a short synthetic route (2*S*,3*S*)-4-fluorosphingosine and 4-fluoroceramide, the fluorinated analogues of the natural products, D-*erythro*-sphingosine and ceramide, have been prepared. The key step of the synthetic sequence is an asymmetric aldol reaction of (*Z*)-2-fluorohexadec-2-enal, prepared in three steps from tetradecanal, with an enantiopure *N*-protected iminoglycinate. Deprotection of the imino function and reduction of the ester group led to the 4-fluorosphingosine, which on acetylation with stearoyl chloride gave 4-fluoroceramide. After careful HPLC purification of the latter compound its phase behavior was investigated by Langmuir film balance technique and compared to that of natural ceramide. While the isotherms are quite similar in shape, they differ significantly in the starting point of increasing film pressure (56 or 67 Å^2^/molecule) and in the film collapse pressure (38 or 56 mN/m) for ceramide and 4-fluoroceramide, respectively. Moreover, the hysteresis curves are very different. While consecutive isothermic compression – expansion cycles are reversible for the 4-fluoro derivative, substantial substance loss into the subphase or irreversible formation of multi-layers was observed for natural ceramide.

**Conclusions:**

Asymmetric aldol reaction proved to be successful for the preparation of enantiopure 4-fluoroceramide. Surface/pressure isotherms and hysteresis curves of ceramide and its 4-fluoro derivative showed that the presence of fluorine leads to stronger intermolecular interactions between the hydrophobic chains of neighboring molecules, and therefore to increasing stability of the monolayer of 4-fluoroceramide at the air water interface.

## Introduction

**Figure 1:**
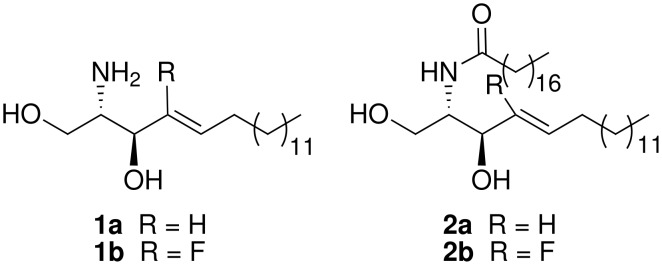
Natural sphingosines **1a**, **2a** and synthesized fluorinated analogues **1b**, **2b**.

Sphingolipids belong to the most important constituents of the membranes of eukaryotic cells. As intermediates in the sphingolipid metabolism, sphingosine (**1a**) and its *N*-octadecanoyl-derivative, ceramide (**1b**) (Figure 1), exhibit a variety of biological functions [1–2]. They play a major role as intracellular signal molecules (second messengers) and mediate signals for essential processes such as cell growth, cell differentiation, cell recognition and apoptosis [3–9]. Moreover, sphingosine is known as an inhibitor of protein kinase C [10–11]. The dynamic balance between ceramide, sphingosine and sphingosine-1-phosphate seems to be decisive for cell growth or apoptosis [12–13]. The specific initiation of apoptosis by suitable derivatives of these signal molecules is discussed as a new method for treatment of numerous diseases [1,14–15], and of cancer in particular [16–18].

A few years ago Herdewijn et al. showed that fluorinated ceramide and dihydroceramide analogues with chain length C_12_ and a fluorine atom instead of the OH group at C(3) exhibit significantly higher apoptosis activity in different cell cultures as compared to their non-fluorinated parent compounds [19]. Furthermore, L-*threo*-3-fluorodihydroceramides with short chain amido groups at C(2) were identified as moderate inhibitors of the dihydroceramide desaturase [20]. Several other fluorinated C_12_ sphingosine and sphinganine analogues inhibited the sphingosine kinase [21] and the corresponding fluorinated C_18_ derivatives were shown to be inhibitors of the protein kinase C [22]. Recently, a D-*erythro*-1-deoxy-1-fluoroceramide analogue was shown to inhibit the formation of sphingomyeline and glycosylceramide in cultured murine neurons, but only in high concentrations (100 μM) [2]. Moreover, sphingolipids are crucial, e.g. for the function of the skin because they contribute to the formation of the water permeability barrier consisting of a highly organized multilaminar lipid matrix of free fatty acids, cholesterol and ceramides containing additional hydroxyl groups in the sphingosin part and longer fatty acid amide functions [23]. The function of the additional free OH group seems to be the formation of additional hydrogen bridges, which enhance the rigidity of the intercellular lipid aggregates and hence decrease the transepidermal water loss [24–25].

Several of the biological properties of sphingosines and ceramides (e.g. sphingomyelinase activity) were assigned to the OH group in the 3-position. While the primary OH group is functionalized with a carbohydrate, a phosphate, sulfate, etc. the 3-OH group is free for various interactions with other constituents of the cell membrane such as cholesterol or proteins [1,26]. The nature of these interactions among other factors depends on the hydrogen bond donating and hydrogen bond accepting properties of the hydroxyl group. Consequently, placement of electron donating or electron accepting substituents close to this group will modify these properties and hence will change the physical, chemical as well the physiological properties of the fluorinated analogues compared to their natural parents. Recently we have demonstrated the effect of a fluorine substituent in the 4-position on the phase behavior at the air/water interface of diastereomeric enantiopure 2-azido-4-fluoro-3-hydroxystearates [27], the precursors of the enantiomers of both diastereomeric 4-fluoro-4,5-dihydroceramides, which we synthesized recently [28].

We became interested in studying the properties and report in this paper the stereoselective synthesis of (*Z*)-2-amino-4-fluorooctadec-4-ene-1,3-diol (4-fluorosphingosine, **1b**) and (*Z*)-2-octadecanoylamino-4-fluorooctadec-4-ene-1,3-diol (4-fluoroceramide, **2b**) having the D-*erythro*-configuration (2*S*,3*S*) and the *trans*-configured C(4)-C(5) double bond of the natural compounds **1a** and **2a** (Figure 1). Our first investigations on the phase behavior at the air/water interface of 4-fluoroceramide (**2b**) and its non-fluorinated analogue **2a** by Langmuir film balance measurements are also presented.

## Results and Discussion

**Scheme 1:**
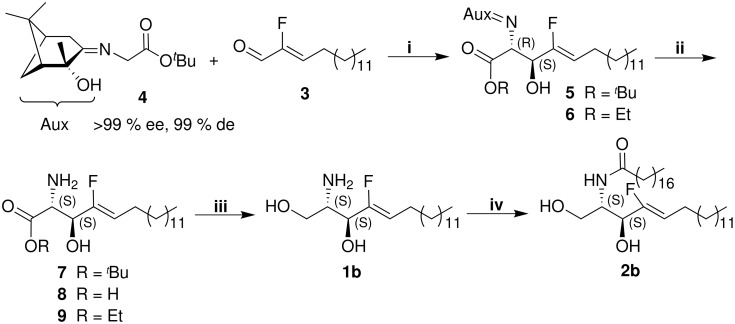
Synthesis of 4-fluorosphingosine (**2b**); Reagents: **i** ClTi(OEt)_3_/Et_3_N, CH_2_Cl_2_, 13 h, 0 °C; **ii** 15% aq citric acid, THF, 68 h, r.t.; **iii** NaBH_4_, EtOH:H_2_O (3:1), 28 h, 0 °C; **iv** C_17_H_35_COCl/50% aq AcONa, 24 h, r.t.

Our synthetic sequence started from (ethoxycarbonylfluoromethyl)triphenylphosphonium bromide and tetradecanal, from which (*Z*)-2-fluorohexadec-2-enal (**3**) was prepared in three steps according to a synthetic route we developed recently for the preparation of long chain α-fluoro-α,β-unsaturated carboxylic acid esters [29] and fluorinated 2,4-dienecarboxylic acid esters [30]. The key step of the synthesis is an asymmetric aldol reaction of the fluorinated aldehyde **3** with the enantiopure iminoglycinate **4** (Scheme 1). The latter building block has already been used for the preparation of several γ-fluoro-α-amino acids [31]. This methodology, utilizing the corresponding ethyl iminoglycinate instead of **4**, was previously applied for the synthesis of natural D-*erythro*-sphingosine (**1a**) [32], deuterium and tritium labeled sphingosines [33] and various other non-fluorinated sphingosine, sphinganine and phytosphingosine derivatives [34].

The aldol reaction was carried out with a small excess of the iminoglycinate **4** (1.1 equiv) and in the presence of 1.6 equiv ClTi(OEt)_3_ [35] and 2.0 equiv of Et_3_N. After 13 h at 0 °C the reaction provided the desired *tert*-butyl imino acid ester **5** as a mixture with the ethyl imino acid ester **6** (formed due to a partial transesterification of **4** with the titanium reagent) and four non-identified compounds (among them most likely diastereomers of the title compounds) in a ratio of 57:28:7:1:2:5, respectively, as detected by ^19^F NMR spectra. The ratio between the major products **5** and **6** was determined to be 65:35. The starting aldehyde **3** (12% from the crude product) was also found in the isolated mixture. Extension of the reaction time or increasing the reaction temperature to r.t. to achieve complete conversion of **3** was not successful. In this case, according to the ESI-MS spectra, besides the iminoglycinate **4**, its analogue with ethoxy group as well 2-hydroxypinan-3-one were also present in the crude product. During the purification by column chromatography a partial cleavage of the C(2)-C(3) bond (retro-aldol reaction) and partial elimination of the auxiliary occurred. Therefore no pure compounds were isolated (for analytical investigations an 88:12 mixture of compounds **5** and **6** was applied) and the crude product was used in the following reaction without purification.

**Scheme 2:**
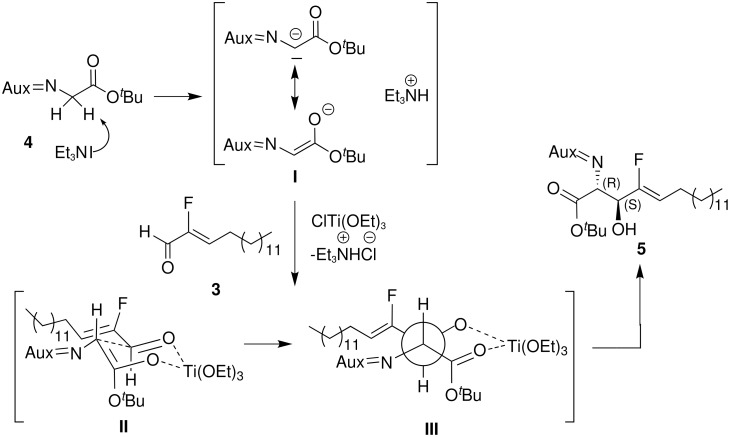
Mechanism of the aldol reaction.

For both major products, **5** and **6**, the D-*erythro*-configuration of the stereogenic centers is most probable, considering the reaction mechanism we propose in Scheme 2. Moreover, the ^3^*J*_H,H_-coupling constants between the protons at C(2) and C(3), which were determined to be 7.8 Hz and 7.7 Hz for **5** and **6**, respectively, support this assignment. The *Z*-configuration of the double bond was determined mainly by the ^3^*J*_H,F_-coupling constants between the fluorine atom and the vinylic proton and between the fluorine and the proton next to the OH group in the ^1^H NMR. For the *tert*-butyl imino acid ester **5** *J* = 37.6 Hz and 19.8 Hz, respectively, were found. The appropriate coupling constants in case of the ethyl imino acid ester **6** were determined from the ^19^F NMR (because the signals do overlap in ^1^H NMR) to be 38.3 Hz and 18.7 Hz, respectively.

**Figure 2:**
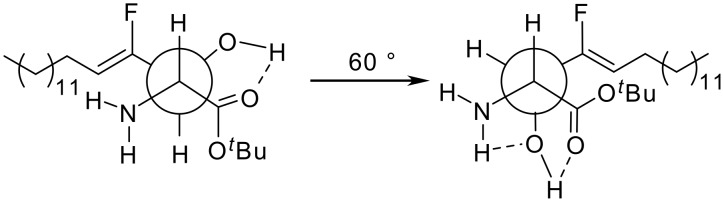
Favorable conformations of the *tert*-butyl amino acid ester **7**.

The crude product obtained from the aldol reaction was partially deprotected with 15% aq solution of citric acid for 68 h at r.t. ^19^F NMR analysis of the crude product showed the formation of three major compounds, which were identified as the *tert*-butyl amino acid ester **7**, the carboxylic acid **8** and the ethyl amino acid ester **9** in a ratio of 31:25:44. Because of partial decomposition on silica gel compounds **8** and **9** could not be isolated in pure form. The D-*erythro*-configuration was confirmed by the ^3^*J*_H,H_-coupling constants for the proton at C(2) in ^1^H NMR, which are 5.0 Hz for compound **7** and 4.7 Hz for compound **9**. These values correlate well with the corresponding coupling constant of 5.0 Hz, given for the non-fluorinated analogue [32]. This small coupling constant is probably due to the fact that the chain in the head group area is not zigzag arranged. More favored is the *gauche* conformation, which is stabilized by intramolecular hydrogen bonds between the CO, OH and NH_2_ groups, as shown in Figure 2. The *trans*-configuration of the double bond was confirmed by the ^3^*J*_H,F_-coupling constants, 38.0 Hz for compound **7** and 38.1 Hz for compound **9**.

The crude product mixture of the hydrolysis was used in the following reduction without purification. The reduction with 8.0 equiv excess of NaBH_4_ at 0 °C for 28 h gave the desired 4-fluorosphingosine (**1b**) and a small amount of a non-identified product (ratio 97:3 ^19^F NMR). Also 24% of non-converted *tert*-butyl ester **7** and traces of non-identified products were present in the reaction mixture. Because of the observed instability of **1b**, no chromatographic purification was performed. The crude product was treated with stearoyl chloride (1.3 equiv) in a mixture of THF and 50% aq solution of AcONa. According to the ^19^F NMR spectra the *N*-octadecanoyl derivative of compound **7**, a non-identified trace compound and the desired 4-fluoroceramide (**2b**) were present in a ratio 27:1:72, respectively. Other non-identified products (together 34%) with ^19^F NMR chemical shifts between δ –115.0 ppm and δ –124.3 ppm were also detected in the mixture. A part (0.1 g) of the crude product was purified by HPLC (CHCl_3_:MeOH, 98:2) in order to isolate 4-fluoroceramide (**2b**) as a white solid in 76% purity and 30% yield. For analytical investigations the described substances were prepared similarly and purified by column chromatography giving compounds with 61–99% purity (for details see Supporting Information File 1). For the investigations of the phase behavior of 4-fluoroceramide (**2b**) at the air/water interface a >99% pure compound was used.

The diastereoselectivity of the aldol reaction, described above, is controlled by the formation of a titanium enolate, which may follow the mechanism we propose in Scheme 2.

The iminoglycinate **4** is deprotonated with Et_3_N to the resonance stabilized anion **I**. ClTi(OEt)_3_ coordinates the carbonyl oxygens of **4** and **3** in a six membered Zimmermann-Traxler transition state **II**. The resulting structure of the titanium alcoholate **III** shows the *erythro*-configuration of the *tert*-butyl amino acid ester **7** and its derivatives **8** and **9**. The absolute configuration (2*S*,3*S*) of the products is controlled by the chiral auxiliary.

In recent years several studies on cell membrane lipid models suggested that ceramide could act indirectly as a messenger by modulation of membrane properties. The membrane lipids (mostly sphingomyelin) together with cholesterol are organized in small domains, known as rafts, stabilized by hydrogen bonds among the polar head groups and van der Waals interactions of the hydrophobic chains. The presence of lipid domains is thought to be involved in receptor-mediated signal transduction. Due to its ability to form large hydrogen bonded networks, because its polar head groups can act both as acceptor and as a donor, ceramide, when added or generated *in situ* in the membrane, can segregate from the other lipids and cause coalescence of the small lipid raft domains to give highly ordered ceramide-enriched domains. Moreover, due to the small size of its polar head group ceramide can displace the raft-associated cholesterol [36–39].

**Figure 3:**
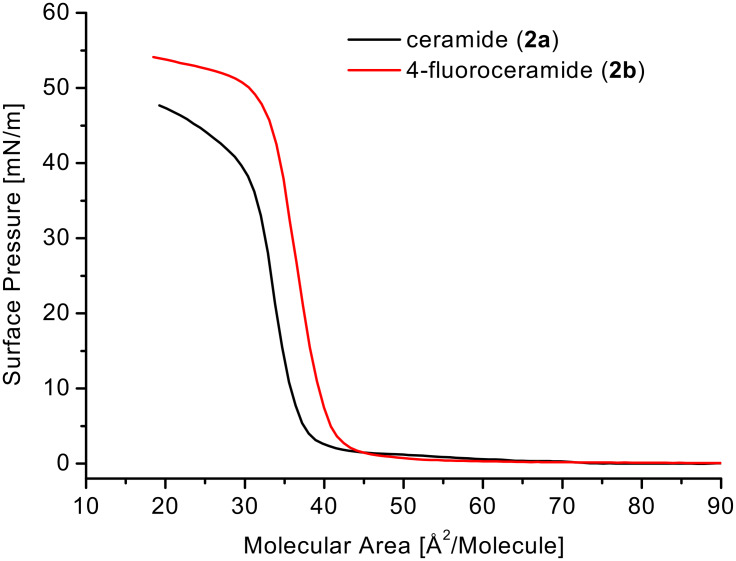
π–A Isotherms of ceramide (**2a**) and 4-fluoroceramide (**2b**) at 20 °C (80 cm^2^/min compression velocity).

In this context, having the fluorinated analogue **2b** of ceramide in hand, we were interested to compare its phase behavior at the air/water interface to that of the corresponding non-fluorinated compound **1b** in order to study the effect of the fluorine atom on the arrangement of the molecules at the water surface. Using Langmuir film balance, the molecular area/surface pressure isotherms (π–A isotherms) shown in Figure 3 were measured at 20 °C.

**Figure 4:**
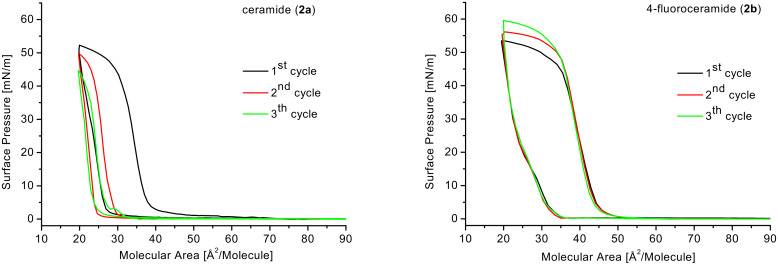
Cycles of compression and expansion for ceramide (**2a**) and 4-fluoroceramide (**2b**).

The curve progression is very similar for both compounds and also correlates with the π–A isotherms of C_18_ ceramide and some of its analogues measured from Löfgren and Pascher at 22 °C [40], as well with the π–A isotherms of the 4-position fluorinated dihydroceramide analogues [41] and of C_16_ ceramide [42]. Both isotherms run over a large interval parallel to the X axis. At 56 Å^2^/molecule for **2a** and at 67 Å^2^/molecule for **2b** the surface pressure starts to increase. In the case of fluorinated ceramide **2b** the film collapses at substantially higher pressure (56 mN/m) then **2a** (38 mN/m), which refers to an increasing stability of the film due to the presence of fluorine. The change of the temperature to 10 °C or 30 °C does not cause any dramatically different curve course for both substances. But a significant difference in the molecules behavior is observed while measuring three consecutive isotherm cycles of compression and expansion (Figure 4).

The isotherm of **2b** shows only a slight shift of the compression curves to higher pressures while the curves of **2a** move significantly to smaller molecular area after every cycle. Thus, there is no loss of substance into the subphase in case of the fluorinated compound **2b**, while molecules of **2a** go partly into the subphase or form multi-layers irreversibly. It seems that the molecules of 4-fluoroceramide (**2b**) interact more strongly with their hydrophobic parts due to the presence of fluorine, which might form intermolecular hydrogen bridges to the vinylic proton of the next molecule. Similar effects were observed in compressed monolayers of ethyl (*Z*)-2-fluorooctadec-2-enoate [29] and ethyl (2*E*,4*Z*)-4-fluorooctadeca-2,4-dienoate [43]. Moreover, a very short C–H···F–C distance (2.30 Å) was observed in crystalline state for (*Z*)-2-amino-4-fluorododec-4-enecarboxylic acid [44].

## Conclusion

In this paper a short diastereo- and enantioselective synthetic route was presented for the preparation of the first analogues **1b** and **2b**, fluorinated in 4-position, of the natural signaling molecules sphingosine (**1a**) and ceramide (**2a**) with the required D-*erythro*-configuration (2*S*,3*S*) of the stereogenic centers and a *Z* configured C(4)-C(5) double bond. It is noteworthy that the presence of both, the fluorine atom and the ester moiety, close to the C(2)-C(3) bond decreases considerably the stability of this bond due to the strong electron withdrawing power of both substituents, which finally leads to a cleavage of the bond during chromatographic purification or at elevated temperature, as was observed in case of imino acid esters **5** and **6**. This might hold for the overall instability and sensitivity against several factors of the fluorinated analogues reported here comparing to their non-fluorinated parent compounds. This complicates the synthesis and purification of these compounds.

By Langmuir film balance measurements we demonstrated that the presence of the fluorine in 4-fluoroceramide (**2b**) leads to stronger intermolecular interactions between the hydrophobic chains of neighboring molecules, comparing to the non-fluorinated parent compounds, and therefore to higher stability of the monolayer formed at the air/water interface. This unique behavior of the 4-fluoroceramide molecules provides the basis for further development of the morphology of the monolayer and possible formation of multi-layers, as well as for biological investigations such as the expected apoptosis activity of **2b**.

## Supporting Information

File 1 General methods, synthesis of the compounds and spectroscopic structure assignment.
