# Supplementary material for: Stereoselective synthesis of (2S,3S,4Z)-4-fluoro-1,3-dihydroxy-2-(octadecanoylamino)octadec-4-ene, [(Z)-4-fluoroceramide], and its phase behavior at the air/water interface
Source: Beilstein J Org Chem. 2008 Apr 25;4:12. doi: 10.3762/bjoc.4.12 (PMC2486483; doi:10.3762/bjoc.4.12)
Supplement: File 1 — General methods, synthesis of the compounds and spectroscopic structure assignment. [file Beilstein_J_Org_Chem-04-12-s001.doc]

**Stereoselective synthesis of (2*S*,3*S*,4*Z*)-4-fluoro-1,3-dihydroxy-2-(octadecanoylamino)octadec-4-ene, [(*Z*)-4-fluoroceramide], and its phase behavior at the air/water interface**

Gergana S. Nikolova and Günter Haufe*

Address: Organisch-Chemisches Institut, Westfälische Wilhelms-Universität Münster, Corrensstr. 40, D-48149 Münster, Germany

Email: Gergana S. Nikolova – [nikolova@uni-muenster.de](mailto:nikolova@uni-muenster.de); Günter Haufe* – [haufe@uni-muenster.de](mailto:haufe@uni-muenster.de)

* Corresponding author

Description: General methods, synthesis of the compounds and spectroscopic structure assignment.

**Experimental**

**General Methods**

Melting points are uncorrected and were taken on a Reichert microscope. Column chromatography was performed on silica gel 60 (0.063-0.200 mm) from Merck. For HPLC a Merck-Hitachi system D‑7000 with a pump L‑7150 and a refractometer Shodex RI‑72 were used. The preparative HPLC was carried out on a Macherey-Nagel column SP 250/21 Nucleosil 50‑7D‑700. NMR spectra were recorded on a Bruker ARX300 (^1^H NMR, 300 MHz; ^13^C NMR, 75 MHz; ^19^F NMR, 282 MHz) and a Varian Inova (1D ^1^H NMR, 500 MHz and ^13^C NMR, 126 MHz, 2D ^1^H,^1^H gCOSY ^1^H,^13^C gHMBC, ^1^H,^13^C gHSQC) spectrometers using TMS (^1^H), CDCl_3_ (^13^C) and CFCl_3_ (^19^F) as internal standards. The ESI-MS spectra were recorded on a Bruker Daltronics Micro Tof. Specific optical rotation [*α*]_D_^20^ was measured with a Perkin-Elmer 341 polarimeter in units of deg·cm^3^dm^-1^·g^-1^. All air‑ and moisture-sensitive reactions were performed under argon atmosphere applying the standard Schlenck-technique. Solvents were purified and dried where necessary using literature methods. Surface pressure-Molecular area (π‑A) isotherms were recorded on a 622 type NIMA Teflon trough (Coventry, U. K.) with a total surface area of 1300 cm^2^. The surface pressure was measured with a Wilhelmy plate system. Ultra pure water (5.5 pH) was obtained from a Millipore system comprising reverse osmosis, ion exchange and 0.22 μm ultra filtration cartridges. The ceramides were dissolved in CDCl_3_:CD_3_OD, 9:1 (HPLC grade), purchased from Merck (Darmstadt, Germany), to have solutions with 1.3·10^-3^ mol/L concentration (0.750 mg/ml for **2a**, 0.785 mg/ml for **2b**). Monolayer was prepared by spreading the appropriate solution onto the water surface using a 100 μL syringe. After 10 min of solvent evaporation the monolayer was compressed with a velocity of 80 cm^2^/min.

**Synthesis of the compounds and spectroscopic structure assignment**

***tert*-Butyl and Ethyl (2*R*,3*S*,4*Z*)‑4-Fluoro-3-hydroxy-2-(2-hydroxy-2,6,6-trimethylbicyclo[3.1.1]­heptan-3-ylideneamino)octadec-4-ene acetates (5 and 6).** To an ice-cold solution of *tert*-butyl (1*R*,2*R*,5*R*)-(+)‑(2-hydroxy-2,7,7-trimethylbicyclo[3.1.1]heptan‑3-ylideneamino) acetate (**4**) [[[1]](#endnote-2)] (3.35 g, 11.9 mmol, 1.1 equiv, >99% ee, >99% de) in anhydrous CH_2_Cl_2_ (7 mL) under argon atmosphere were added successively ClTi(OEt)_3_ [[[2]](#endnote-3)] (3.78 g, 17.3 mmol, 1.6 equiv) in anhydrous CH_2_Cl_2_ (7 mL), (*Z*)‑2‑fluorohexadec‑2‑enal (**3**) [[[3]](#endnote-4)] (2.78 g, 10.8 mmol) in anhydrous CH_2_Cl_2_ (6 mL) and anhydrous Et_3_N (3 mL, 2.19 g, 21.6 mmol, 2.0 equiv). The reaction mixture was stirred for 13 h at 0°C and then quenched with brine (120 mL). After the aqueous phase was extracted with EtOAc (4 × 80 mL) the combined organic extracts were dried over MgSO_4_ and concentrated under vacuum. ^19^F NMR analysis of the crude product showed the formation of the *tert*-butyl imino acid ester **5**, the ethyl imino acid ester **6** and four non-identified compounds (among them probably diastereomers of the title compounds) in a 57:28:7:1:2:5 ratio at δ −126.91 ppm, δ −127.31 ppm, δ −127.36 ppm, δ −128.26 ppm, δ −128.72 ppm, and δ −129.26, respectively. The aldehyde **3** (12% of the crude product) was also found in the isolated mixture. According to the ESI‑MS spectra iminoglycinate **4** and its analogue with ethoxy group **6**, as well 2-hydroxypinan-3-one were also present in the crude product. During the purification by column chromatography (Et_2_O:hexane, 7:3) partial cleavage of the C(2)-C(3)-bond (retroaldol reaction) and partial elimination of 2-hydroxypinan-3-one occurred. Therefore the crude product was used in the following deprotection step without purification. Yield: 5.24 g (crude). In a similar experiment at r.t. a mixture of *tert*-butyl imino acid ester **5** (70%, ^19^F NMR), ethyl imino acid ester **6** (10%), aldehyde **3** (16%) and a non-identified product (4%) was isolated after column chromatography. This mixture was characterized spectroscopically. *tert*-Butyl imino acid ester **5**. ^1^H NMR (300 MHz, CDCl_3_) δ 0.88 (t, ^3^*J*_H,H_ = 6.0 Hz, 3 H, CH_3_), 0.90 (s, 6 H, 2CH_3_), 1.23−1.28 (m, 24 H, CH_2_), 1.33 (s, 3 H, CH_3_), 1.39 (s, 3 H, CH_3_), 1.43 (s, 9 H, 3CH_3_), 1.48 (d, ^3^*J*_H,H_ = 5.6 Hz, 1 H, CH_2_ *_anti_*), 2.02−2.08 (m, 2 H, 2CH), 2.28−2.38 (m, 2 H, CH_2 syn_), 2.61−2.63 (AB, 2 H, CH_2_), 4.08 (br. s, 1 H, OH), 4.32 (d, ^3^*J*_H,H_ = 7.8 Hz, 1 H, 2-CH), 4.55 (dd, ^3^*J*_H,F_ = 19.8 Hz, ^3^*J*_H,H_ = 7.9 Hz, 1 H, 3-CH), 4.90 (dt, ^3^*J*_H,F_ = 37.6 Hz, ^3^*J*_H,H_ = 7.5 Hz, 1 H, 5‑CH); ^13^C NMR (75 MHz, CDCl_3_) δ 14.1 (q), 22.7 (t), 22.8 (q), 25.3 (q), 27.3 (q), 28.0 (q), 28.1 (t), 28.4, 29.1, 29.3, 29.4, 29.6, 29.6 (all t), 31.9 (t), 33.4 (t), 38.4 (d), 38.5 (s), 50.0 (d), 64.8 (d, C-2), 72.0 (dd, ^2^*J*_C,F_ = 27.6 Hz, C-3), 77.1 (s), 82.4 (s), 109.8 (dd, ^2^*J*_C,F_ = 12.5 Hz, C-5), 156.1 (d, ^1^*J*_C,F_ = 255.2 Hz, C‑4), 169.5 (s), 180.8 (s); ^19^F NMR (282 MHz, CDCl_3_) δ −127.1 (dd, ^3^*J*_F,H_ = 38.2 Hz, ^3^*J*_F,H_ = 20.4 Hz); ESI-MS m/z (%): 560 (59) [M + Na]^+^, 538 (100) [M + H]^+^, 482 (26) [538 – C_4_H_8_^+^] 464 (8). Ethyl imino acid ester **6**. Most of the signals for **6** in ^1^H and ^13^C NMR are overlapping with the signals of the *tert*-butyl imino acid ester **5**. ^1^H NMR (300 MHz, CDCl_3_) δ 1.28 (t, 3 H, CH_3_), 4.12 (q, ^3^*J*_H,H_ = 7.2 Hz, 2 H, CH_2_), 4.42 (d, ^3^*J*_H,H_ = 7.7 Hz, 1 H, 2-CH); ^13^C NMR (75 MHz, CDCl_3_) δ 14.2 (q), 61.4 (t), 64.3 (d, C-2). ^19^F NMR (282 MHz, CDCl_3_) δ −127.4 (dd, ^3^*J*_F,H_ = 38.3 Hz, ^3^*J*_F,H_ = 18.7 Hz); ESI-MS m/z (%): 532 (17) [M + Na]^+^, 510 (21) [M + H]^+^.

***tert*‑Butyl and Ethyl (2*R*,3*S,*4*Z*)‑2‑Amino-4‑fluoro-3‑hydroxyoctadec-4‑enoates (7 and 9).** The crude product (5.00 g, 9.3 mmol, based on the molecular weight of the main component **5** of the mixture) obtained in the aldol reaction described above, was dissolved in THF (16 mL) and 15% aq solution of citric acid (64 mL) and stirred at r.t. for 68 h. After evaporation of the solvent under vacuum the aqueous phase was neutralized with Na_2_CO_3_ to pH=7 and extracted with Et_2_O (4 × 60 mL). The combined organic layer was dried over Na_2_SO_4_ and concentrated under vacuum. ^19^F NMR analysis of the crude product showed the formation of three main compounds: *tert*-butyl amino acid ester **7**, 2‑amino-4‑fluoro-3‑hydroxyoctadec-4‑enoic acid (**8**) and the ethyl amino acid ester **9** in a 31:25:44 ratio, respectively. Because of partial decomposition of the title compounds on silica gel, the crude product was used in the following reaction without purification. Yield: 4.00 g (crude). By a similar experiment compounds **7** and **9** were isolated after column chromatography (Et_2_O:MeOH, 99:1), followed by recrystallization from pentane, as white solids in 99% and 61% (a mixture of **7** and **9** in ratio 39:61) purity, respectively, and spectroscopically characterized. *tert*‑Butyl (2*R*,3*S*,4*Z*)-2‑amino-4‑fluoro-3‑hydroxy­octadec-4‑enoate (**7**). Mp 42–44 °C (pentane); [*α*]_D_^20^ = +19.7 (*c* = 1.00, CHCl_3_); ^1^H NMR (300 MHz, CDCl_3_) δ 0.88 (t, ^3^*J*_H,H_ = 6.7 Hz, 3 H, CH_3_), 1.23−1.30 (m, 22 H, CH_2_), 1.46 (s, 9 H, CH_3_), 2.07 (m, ^3^*J*_H,H_ = 6.6 Hz, 2 H, 6-CH_2_), 2.44 (br s, 1 H, OH), 3.61 (d, ^3^*J*_H,H_ = 5.0 Hz, 1 H, 2-CH), 4.38 (dd, ^3^*J*_H,F_ = 13.2 Hz, ^3^*J*_H,H_ = 5.2 Hz, 1 H, 3-CH), 4.90 (dt, ^3^*J*_H,F_ = 38.0 Hz, ^3^*J*_H,H_ = 7.6 Hz, 1 H, 5‑CH); ^13^C NMR (75 MHz, CDCl_3_) δ 14.1 (q), 22.7 (t), 23.3 (dt, ^3^*J*_C,F_ = 4.3 Hz, C-6), 28.0 (q), 29.2, 29.3, 29.4, 29.6, 29.6 (all t), 31.9 (t), 57.5 (d, C-2), 70.3 (dd, ^2^*J*_C,F_ = 31.4 Hz, C‑3), 82.1 (s), 108.6 (dd, ^2^*J*_C,F_ = 12.5 Hz, C‑5), 156.7 (ds, ^1^*J*_C,F_ = 256.7 Hz), 171.6 (s); ^19^F NMR (282 MHz, CDCl_3_) δ −123.2 (dd, ^3^*J*_F,H_ = 38.2 Hz, ^3^*J*_F,H_ = 13.2 Hz); ESI-MS m/z (%): 410 (36) [M + Na]^+^, 388 (100) [M + H]^+^, 332 (100), 314 (57), 294 (8), 270 (22) (daughters of 388 (31)). Ethyl (2*R*,3*S*,4*Z*)-2‑amino-4‑fluoro-3‑hydroxyoctadec-4‑enoate (**9**). ^1^H NMR (300 MHz, CDCl_3_) δ 0.88 (t, ^3^*J*_H,H_ = 6.8 Hz, 3 H, CH_3_), 1.23−1.29 (m, 22 H, CH_2_), 1.28 (t, ^3^*J*_H,H_ = 7.1 Hz, 3 H, CH_3_), 2.07 (m, ^3^*J*_H,H_ = 6.8 Hz, 2 H, 6-CH_2_), 2.41 (br s, 1 H, OH), 3.69 (d, ^3^*J*_H,H_ = 4.7 Hz, 1 H, 2-CH), 4.20 (q, ^3^*J*_H,H_ = 7.2 Hz, 2 H, CH_2_), 4.38 (dd, ^3^*J*_H,F_ = 13.5 Hz, ^3^*J*_H,H_ = 5.6 Hz, 1 H, 3-CH), 4.89 (dt, ^3^*J*_H,F_ = 38.1 Hz, ^3^*J*_H,H_ = 7.5 Hz, 1 H, 5‑CH); ^13^C NMR (75 MHz, CDCl_3_) δ 14.05 (q), 14.1 (q), 22.7 (t), 23.3 (dt, ^3^*J*_C,F_ = 3.7 Hz, C-6), 29.2, 29.2, 29.3, 29.4, 29.6, 29.6 (all t), 31.9 (t), 57.1 (d, C-2), 61.3 (t), 70.4 (dd, ^2^*J*_C,F_ = 30.9 Hz, C‑3), 108.8 (dd, ^2^*J*_C,F_ = 12.5 Hz, C‑5), 156.5 (ds, ^1^*J*_C,F_ = 255.3 Hz), 172.7 (s); ^19^F NMR (282 MHz, CDCl_3_) δ −124.0 (dd, ^3^*J*_F,H_ = 38.1 Hz, ^3^*J*_F,H_ = 13.1 Hz); ESI-MS m/z (%): 360 (100) [M + H]^+^, 342 (100), 322 (2), 314 (2), 298 (4), 268 (12) (daughters of 360 (18)). 2‑Amino-4‑fluoro-3‑hydroxyoctadec-4‑enoic acid (**8**). ^19^F NMR (282 MHz, CDCl_3_) δ −123.4 (dd, ^3^*J*_F,H_ = 35.1 Hz, ^3^*J*_F,H_ = 18.4 Hz); ESI-MS m/z (%): 332 (12) [M + H]^+^, 314 (57), 294 (14), 276 (14), 270 (18) (daughters of 332 (100)).

**(2*S*,3*S*,4*Z*)‑2-Amino-4-fluorooctadec-4-ene-1,3-diol (1b) (4‑Fluorosphingosine)**. The crude product (3.60 g, 9.3 mmol, based on the molecular weight of the main component **7** of the mixture) obtained from the hydrolysis described above, was dissolved in EtOH:H_2_O (3:1) (80 mL). NaBH_4_ (1.30 g, 34.4 mmol, 8.0 equiv) was added portion wise at 0 °C. The aqueous mixture was stirred for 28 h at 0°C and then quenched with water. The new phase was extracted four times with CH_2_Cl_2_ and twice with CH_2_Cl_2_/MeOH mixture. The combined organic extracts were dried over NaSO_4_ and concentrated under vacuum. ^19^F NMR analysis of the crude product showed the formation of the titled compound **1b** and a non-identified product (δ −125.5 ppm) in a 97:3 ratio, as well as 24% of non-converted *tert*-butyl ester **7** and other non-identified products with chemical shifts between δ −113.7 ppm and δ −120.5 ppm (together 46%, ^19^F NMR). Because of the observed instability of **1b**, the crude product was used in the following reaction without purification. Yield: 3.03 g (crude). By a similar experiment compound **1b** was isolated after column chromatography (CHCl_3_:MeOH:H_2_O, 13:6:1) as a white solid and spectroscopically characterized as a mixture with 30% of a non-identified product. 4‑Fluorosphingosine (**1b**). ^1^H NMR (500 MHz, CD_3_OD) δ 0.90 (t, ^3^*J*_H,H_ = 6.9 Hz, 3 H, CH_3_), 1.29−1.34 (m, 22 H, CH_2_), 1.37-1.43 (m, 2 H, CH_2_), 2.05-2.17 (m, 2 H, CH_2_), 2.97-3.04 (m, 1 H, 2-CH), 3.71 (dd, ^2^*J*_H,H_ = 11.2 Hz, ^3^*J*_H,H_ = 3.5 Hz, 1 H, 1‑CH_2_), 3.59 (dd, ^2^*J*_H,H_ = 11.2 Hz, ^3^*J*_H,H_ = 6.7 Hz, 1 H, 1‑CH_2_), 4.05 (dd, ^3^*J*_H,F_ = 17.6 Hz, ^3^*J*_H,H_ = 6.6 Hz, 1 H, 3-CH), 4.96 (dt, ^3^*J*_H,F_ = 37.9 Hz, ^3^*J*_H,H_ = 7.5 Hz, 1 H, 5‑CH); ^13^C NMR (126 MHz, CD_3_OD) δ 14.8 (q), 24.1 (t), 24.6 (dt, ^3^*J*_C,F_ = 4.3 Hz, C-6), 30.7, 30.8, 30.9, 31.1, 31.1, 31.1 (all t), 33.4 (t), 55.6 (d, C-2), 63.4 (t, C‑1), 72.6 (dd, ^2^*J*_C,F_ = 29.4 Hz, C‑3), 109.9 (dd, ^2^*J*_C,F_ = 12.7 Hz, C‑5), 159.3 (ds, ^1^*J*_C,F_ = 256.5 Hz, C‑4); ^19^F NMR (282 MHz, CD_3_OD) δ −125.3 (dd, ^3^*J*_F,H_ = 37.7 Hz, ^3^*J*_F,H_ = 17.9 Hz); The assignment of the signals was confirmed by 2D ^1^H,^1^H gCOSY, ^1^H,^13^C gHMBC, and ^1^H,^13^C gHSQC NMR spectra. ESI-MS m/z (%): 635 (4) [2M + H]^+^, 318 (100) [M + H]^+^, 300 (11), 270 (1).

**(2*S*,3*S,*4*Z*)-4-Fluoro-1,3-dihydroxy-2-(octadecanoylamino)-octadec-4-ene (2b) (4-Fluoro­ceramide).** To a suspension of the crude product (2.60 g, 8.2 mmol, based on the molecular weight of the main component **1b** of the mixture) obtained from the reduction described above, in THF (41 mL), a 50% aq solution of AcONa (25 mL) and stearoyl acid chloride (3.6 g, 10.7 mmol, 1.3 equiv) were added. After stirring for 24 h at r.t. the reaction mixture was poured into brine (60 mL). The phases were separated and the aqueous layer was extracted with THF (3 × 100 mL). The combined organic phases were washed with brine (2 × 300 mL), dried over NaSO_4_, filtered through a short pad of silica gel and concentrated under vacuum. ^19^F NMR analysis of the crude product showed the formation of the *N-*octadecanoyl derivative of compound **7**, a non-identified product (δ −124.8 ppm) and the titled compound **2b** in a 27:1:72 ratio, respectively, as well as other non-identified products with chemical shifts between δ −115.0 ppm and δ −124.3 ppm. Yield: 6.70 g (crude). A fraction of the crude product (0.10 g) was purified by HPLC (CHCl_3_:MeOH, 98:2) and 4-fluoroceramide (**2b**) was isolated as a white solid in 76% purity (^19^F NMR). Yield: 0.03 g (30%, **2b**). By a similar experiment compound **2b** was isolated in 86% purity after HPLC (CHCl_3_:MeOH, 98:2) and spectroscopically characterized. For the investigations on the phase behavior of **2b** at the air/water interface a >99% pure compound obtained by repeated HPLC was used. 4-Fluoroceramide (**2b**). Mp 100–102 °C (CHCl_3_/MeOH, 99% pure **2b**), ^1^H NMR (300 MHz, CDCl_3_:CD_3_OD, 9:1) δ 0.88 (br t, ^3^*J*_H,H_ = 6.2 Hz, 6 H, CH_3_), 1.20−1.33 (m, 22 H, CH_2_), 1.56−1.64 (m, 2 H, CH_2_), 2.05−2.10 (m, 2 H, CH_2_), 2.21 (br t, ^3^*J*_H,H_ = 7.5 Hz, 2 H, CH_2_), 3.67 (dd, ^2^*J*_H,H_ = 11.2 Hz, ^3^*J*_H,H_ = 3.8 Hz, 1 H, 1‑CH_2_), 3.87 (dd, ^2^*J*_H,H_ = 11.1 Hz, ^3^*J*_H,H_ = 4.0 Hz, 1 H, 1-CH_2_), 4.00 (br s, 2 H, OH), 4.02−4.07 (br m, 1 H, 2‑CH), 4.20 (dd, ^3^*J*_H,F_ = 15.2 Hz, ^3^*J*_H,H_ = 6.1 Hz, 1 H, 3‑CH), 4.93 (dt, ^3^*J*_H,F_ = 38.0 Hz, ^3^*J*_H,H_ = 7.5 Hz, 1 H, 5‑CH), 7.39 (d, ^3^*J*_H,H_ = 6.8 Hz, 1 H, NH); ^13^C NMR (75 MHz, CDCl_3_:CD_3_OD, 9:1) δ 13.6 (q) 22.3 (t), 23.0 (dt, ^3^*J*_C,F_ = 5.3 Hz, C-6), 25.5 (t), 29.0, 29.0, 29.1, 29.2, (all t), 31.6 (t), 36.3 (t), 52.4 (d, C-2), 61.2 (t, C‑1), 70.4 (dd, ^2^*J*_C,F_ = 32.3 Hz, C‑3), 108.0 (dd, ^2^*J*_C,F_ = 13.6 Hz, C‑5), 159.4 (ds, ^1^*J*_C,F_ = 260.6 Hz, C‑4), 174.5 (s); ^19^F NMR (282 MHz, CDCl_3_:CD_3_OD, 9:1) δ −125.0 (dd, ^3^*J*_F,H_ = 38.0 Hz, ^3^*J*_F,H_ = 15.0 Hz); ESI-MS m/z (%): 606 (100) [M + Na]^+^, 584 (24) [M + H]^+^.

1. Oguri, T.; Kawai, N.; Shiori, T.; Yamada, S. *Chem. Pharm. Bull.* **1978,** *26,* 803–808. [↑](#endnote-ref-2)
2. Jennings, J. S.; Wardlaw, W.; Way, W. J. R. *J. Chem. Soc.* **1936,** 637–640. doi:10.1039/JR9360000637 [↑](#endnote-ref-3)
3. Nikolova, G. S.; Haufe, G. *Synthesis* **2008,** 527–536. doi:10.1055/s-2008-1032147 [↑](#endnote-ref-4)
